# High diagnostic yield of direct Sanger sequencing in the diagnosis of neuronal ceroid lipofuscinoses

**DOI:** 10.1002/jmd2.12057

**Published:** 2019-09-03

**Authors:** Abdulhakim Jilani, Diana Matviychuk, Susan Blaser, Sarah Dyack, Jean Mathieu, Asuri N. Prasad, Chitra Prasad, Lianna Kyriakopoulou, Saadet Mercimek‐Andrews

**Affiliations:** ^1^ Division of Clinical and Metabolic Genetics, Department of Paediatrics University of Toronto, The Hospital for Sick Children Toronto Ontario Canada; ^2^ Division of Genome Diagnostics, Department of Paediatric Laboratory Medicine The Hospital for Sick Children Toronto Ontario Canada; ^3^ Division of Neuroradiology, Department of Medical Imaging University of Toronto, The Hospital for Sick Children Toronto Ontario Canada; ^4^ Division of Medical Genetics, Department of Pediatrics, IWK Health Centre University of Dalhouise Halifax Nova Scotia Canada; ^5^ Neuromuscular Disease Clinic University of Sherbrooke Quebec Canada; ^6^ Division of Clinical Neurosciences, Department of Paediatrics, Schulich School of Medicine and Dentistry Western University London Ontario Canada; ^7^ Division of Medical Genetics^,^ Department of Paediatrics, Schulich School of Medicine & Dentistry Western University London Ontario Canada; ^8^ Department of Paediatric Laboratory Medicine and Pathobiology University of Toronto Toronto Ontario Canada; ^9^ Genetics and Genome Biology Program, Research Institute The Hospital for Sick Children Toronto Ontario Canada; ^10^ Institute of Medical Sciences University of Toronto Toronto Ontario Canada

**Keywords:** *CLN* genes, developmental regression, epilepsy, neuronal ceroid lipofuscinoses, visual loss

## Abstract

**Background:**

Neuronal ceroid lipofuscinoses are neurodegenerative disorders. To investigate the diagnostic yield of direct Sanger sequencing of the *CLN* genes, we reviewed Molecular Genetics Laboratory Database for molecular genetic test results of the *CLN* genes from a single clinical molecular diagnostic laboratory.

**Methods:**

We reviewed electronic patient charts. We used consent forms and Research Electronic Data Capture questionnaires for the patients from outside of our Institution. We reclassified all variants in the *CLN* genes.

**Results:**

Six hundred and ninety three individuals underwent the direct Sanger sequencing of the *CLN* genes for the diagnosis of neuronal ceroid lipofuscinoses. There were 343 symptomatic patients and 350 family members. Ninety‐one symptomatic patients had molecular genetic diagnosis of neuronal ceroid lipofuscinoses including *CLN1 (PPT1)* (n = 10), *CLN2* (*TPP1*) (n = 33), *CLN3* (n = 17), *CLN5* (n = 7), *CLN6* (n = 10), *CLN7* (*MFSD8*) (n = 10), and *CLN8* (n = 4) diseases. The diagnostic yield of direct Sanger sequencing of *CLN* genes was 27% in symptomatic patients. We report detailed clinical and investigation results of 33 NCL patients. Juvenile onset *CLN1* (*PPT1*) and adult onset *CLN6* diseases were nonclassical phenotypes.

**Conclusion:**

In our study, the diagnostic yield of direct Sanger sequencing was close to diagnostic yield of whole exome sequencing. Developmental regression, cognitive decline, visual impairment and cerebral and/or cerebellar atrophy in brain MRI are significant clinical and neuroimaging denominators to include NCL in the differential diagnosis.

**SYNOPSIS**

The diagnostic yield of direct Sanger sequencing of the *CLN* genes was 27% in symptomatic patients and developmental regression, visual impairment and cerebral and/or cerebellar atrophy in brain MRI are significant clinical and neuroimaging denominators of neuronal ceroid lipofuscinoses.

## 1 INTRODUCTION

Neuronal ceroid lipofuscinoses (NCL) are neurodegenerative, lysosomal storage disorders, characterized by a history of developmental regression in motor and cognitive functions, seizures, visual problems, and early death. The estimated incidence of NCL ranges from 1.3 to 7 per 100 000 live births. NCL are the most common neurometabolic neurodegenerative disease with an estimated prevalence of 1.5 to 9 per million population.^1^, ^2^ The most prevalent NCL are *CLN3* disease (MIM#204200) and *CLN2* (*TPP1*) disease (MIM#204500). The onset of first symptoms varies within the same genetic subtype for the vast majority of NCL.^1^ In three of the subtypes, enzyme activity can be measured in a blood dot spot including cathepsin D (EC 3.4.23.5), encoded by *CTSD* (MIM#116840), palmitoyl‐protein thioesterase (EC 3.1.2.22), encoded by *PPT1* (MIM#600722) and tripeptidylpeptidase 1 (EC 3.4.14.9), encoded by *TPP1* (MIM#607998).^3^ The electron microscopic examination of lymphocytes, skin cells or cells from conjunctival biopsy has been performed to identify lysosomal inclusions, called lipopigments and described as granular osmiophilic, curvilinear and fingerprint profiles Pathogenic variants in 13 different genes are known to cause NCL including *CLN1* (*PPT1*), *CLN2* (*TPP1*), *CLN3* (MIM#607042), *CLN5* (MIM#256731), *CLN6* (MIM#601780), *MFSD8* (*CLN7*) (MIM#611124), *CLN8* (MIM#607837), *CTSD* (*CLN10*), *KCTD7* (MIM#611725), *ATP13A2* (MIM#610513), *CTSF* (MIM#603539), *GRN* (MIM#138945), and *DNAJC5* (MIM#611203).^1^ Treatment is symptomatic for the majority of NCL. Recently, intracerebroventricular infusions of cerliponase alfa for the treatment of *CLN2* (*TPP1*) disease were approved.^4^

In this study, we investigated the diagnostic yield of direct Sanger sequencing of the *CLN* genes for the molecular genetic diagnosis of NCL in our Molecular Genetics Laboratory. This laboratory is one of the reference laboratories providing direct Sanger sequencing for eight *CLN* genes for patients with suspected NCL.

## 2 MATERIALS AND METHODS

Institutional Research Ethics Board (Approval#1000059020) approved this study. We included all individuals, who underwent Sanger sequencing of the *CLN* genes in the Molecular Genetics Laboratory. We entered clinical features, family history and investigation results of all individuals into an Excel Database who had medical records at our institution. If the patients with confirmed molecular genetic diagnosis of NCL were from other centers, we informed physicians for this study, who requested genetic investigations for NCL diagnosis in their patients. We provided a study introduction letter and a release of information consent form to inform patients and families for this study. Patients and families contacted us and signed two consents: (a) consent to enroll to the study; (b) consent for release of information for their physicians. After consents are signed, we sent invitation for Research Electronic Data Capture (REDCap) questionnaire to the physicians, where they entered their patients' clinical information, family history, and investigation results.

From 2004 to 2008, we applied direct Sanger sequencing to six *CLN* genes (*CLN1* (*PPT1*), *CLN2* (*TPP1*), *CLN3*, *CLN5*, *CLN6*, *CLN8*). From 2008 to present we applied direct Sanger sequencing to two additional *CLN* genes (*CLN7* and *CLN10*). All patients underwent direct Sanger sequencing of all seven *CLN* genes, if the laboratory received a request for NCL genetic test using patients' genomic DNA sample. The primers were designed to cover exons and the first 50 bp of introns. The common 1.02 kb deletion in *CLN3* was tested using quantitative PCR. If there was no amplification for exons, quantitative PCR was applied. Synonymous variants were reported if they were previously reported as pathogenic or were predicted to disrupt splicing using Splice Site Finder (SSF), Maximum Entropy Scan (MaxEntScan) (http://genes.mit.edu/burgelab/maxent/Xmaxentscan_scoreseq.html),^5^ Neural Network Splice (NNSPLICE) (Berkeley Drosophila Genome Project, http://www.fruitfly.org/seq_tools/splice.html),^6^ GeneSplicer (https://ccb.jhu.edu/software/genesplicer/).^7^ Only the first five intronic base pair variants were reported, unless there were previously reported pathogenic intronic variants, which were predicted to disrupt splicing in silico analysis tools or predicted to disrupt translation initiation.

We classified variants using Alamut variant interpretation software version 2.7‐2 (http://www.interactive-biosoftware.com) and The American College of Medical Genetics and Genomics (ACMG) variant classification guidelines.^8^ Missense variants were analyzed using Sorting Intolerant From Tolerant (SIFT), Mutation Taster, and Polymorphism Phenotyping V2 (PolyPhen‐2) and conservation in species in silico analysis prediction tools. Intronic or synonymous variants were analyzed using the SSF, MaxEntScan, NNSPLICE and GeneSplicer. We classified a variant as predicted to disrupt splicing if ≥3 of the splicing prediction programs predicted a >10% decrease in the possibility of correct splicing (minus correspondence to decrease). We also searched all variants identified in NCL patients in Genome Aggregation Database (gnomAD) (http://gnomad.broadinstitute.org/about) for their allele frequency in the general population. Homozygous or compound heterozygous variants of unknown significance in a symptomatic patient were interpreted: (A) molecular genetic diagnosis of NCL: (a) if we had clinical, neuroimaging and/or histopathology results of patients in our institution; (b) if the variant was reported previously in NCL patients; (B) no molecular genetic diagnosis of NCL (a) if we did not have clinical, neuroimaging and/or histopathology results of patients with a novel variant.

We utilized the University College London (UCL) NCL Resource Patient Database (http://www.ucl.ac.uk/ncl/) to compare phenotypes, distribution of patients for each subgroup of NCL, as well as publicly available variants in NCL genes.

Fisher's exact test was used for comparisons between patients with molecular genetic diagnosis of NCL and patients with no molecular genetic diagnosis of NCL. All analyses were performed using R statistical software. A *P* value <.05 was considered statistically significant.

## 3 RESULTS

There were 693 individuals underwent direct Sanger sequencing of the *CLN* genes including 343 symptomatic, seven fetuses for prenatal diagnosis, and 343 parents, siblings, spouses, or relatives for carrier test or confirmation of the clinical diagnosis between April 2004 and April 2018. We confirmed molecular genetic diagnosis of NCL in 91 patients from 77 families in 343 symptomatic patients (27% of all symptomatic patients and 22% of all families with symptomatic children).

The number of patients for each gene associated with NCL is depicted in Figure S1. The most common NCL was *CLN2* (*TPP1*) disease and the second most common NCL was the *CLN3* disease. We had clinical information for 33 out of 91 patients with molecular genetic diagnosis of NCL from 27 families including 27 patients at our institution and 6 patients outside of our Institution within Canada. We did not have any clinical information for 58 out of 91 patients with confirmed molecular genetic diagnosis of NCL (35 from Canada and 23 from outside of Canada) for the following reasons: (a) physicians were not able to reach parents due to outdated contact information in their system or they were no longer taking care of the patients; (b) physicians did not want to contact families whose children had passed away so as not to cause further grief; (c) physicians did not respond to our three e‐mail requests; and (d) families were not interested in the study.

All 33 patients presented with a history of developmental delay or developmental regression leading to NCL molecular genetic test request. There were seven patients with *CLN1* (*PPT1*), five patients with *CLN2* (*TPP1*), seven patients with *CLN3* (patients 13 and 15 reported previously [9]), one patient with *CLN5*, four patients with *CLN6*, six patients with *CLN7* (*MFSD8*) and three patients with *CLN8* disease. Their clinical features and investigation results are summarized in Table 1. Eleven patients passed away and their survival period is depicted in Figure S2.

**Table 1:** Clinical features and conjunctival biopsy, neuroimaging, and genetic test results of patients with NCL are listed

Diagnosis/patient study ID number/sex/age/consanguinity	Presenting symptom (age of onset)	Clinical features	Histopathology (CB or SB)/neuroimaging (age)	Genetic test results (reference)
1*/CLN1*(*PPT1*)/23/F/16 yrs^a^/NA	Seizure (7 yrs)	GDD, regression, FTT, seizures (MS, GTCS, focal), aggressive behavior, hallucinations, stereotypic movements	NP/cerebral atrophy, small thalami, increased T2 signal PVWM in MRI (7 yrs)	HMZ known c.674T>C (p.Phe225Ser)^1^ in *CLN1* (*PPT1*)
2*/CLN1*(*PPT1*)/29/M/7 yrs/no	Developmental regression (1 yr)	GDD, cortical blindness, seizures, athetosis, ataxia	Normal (CB)/cerebral atrophy in MRI (2 yrs)	Compound HTZ known c.29T>A (p.Leu10*)^10^/known c.451C>T (p.Arg151*) in *CLN1* (*PPT1*)
3*/CLN1*(*PPT1*)/51/M/18 yrs/NA	Vision problems (<6 yrs)	Cognitive decline, retinitis pigmentosa, hallucinations,	GROP (CB)/cerebral, cerebellar atrophy, thin CC, increased signal intensity in caudate nuclei, putamina, and centrum semiovale in MRI	HMZ known c.223A>C (p.Thr75Pro)^11^ in *CLN1* (*PPT1*)
4*/CLN1*(*PPT1*)/62/F/18 yrs/no	Vision problems (8 yrs)	Cognitive regression, seizures (AbS), retinal dystrophy	NP/cerebral atrophy in MRI (13 yrs)	Compound HTZ known c.451C>T (p.Arg151*)^10^/known c.223A>C (p.Thr75Pro)^11^ in *CLN1* (*PPT1*)
5*/CLN1*(*PPT1*)/76/M/15 yrs/no	Seizures (4 yrs)	GDD, seizures (MS, AbS, GTCS, focal), optic atrophy, cataracts, bull's eye maculopathy, hypertonia	NP/cerebral, cerebellar atrophy, decreased T2 signal in thalamus and globus pallidi T2 signal in MRI (7 yrs)	Compound HTZ known c.541G>T (p.Val181Leu)^12^)/novel c.289_290del (p.Gln97Glyfs*4) in *CLN1* (*PPT1*)
6*/CLN1*(*PPT1*)/112/M/2 yrs 10 mo^a^/no	GDD, regression (1 yr)	GDD, seizures (AbS, GTCS, MS), spasticity, microcephaly	NP/cerebral atrophy, small thalami decreased T2 signal in MRI (2 yrs)	Compound HTZ known c.451C>T (p.Arg151*)^10^/known c.490C>T (p.Arg164*)^12^ in *CLN1* (*PPT1*)
7/*CLN1*(*PPT1*)/168/F/18 yrs/yes	Vision problems (7 yrs)	Developmental regression, aggressive behavior, tremor, RP	GROP (SB)/NP	HMZ known c.223A>C (p.Thr75Pro)^11^ in *CLN1* (*PPT1*)
8/*CLN2*(*TPP1*)/27/M/18 yrs/no	Seizures (3.5 yrs)	GDD, seizures, ataxia	NP/diffuse cerebral and cerebellar atrophy, increased FLAIR signal in MRI (9 yrs)	Compound HTZ known c.509‐1G>C^12^/known c.622C>T (p.Arg208*)^13^ in *CLN2* (*TPP1*)
9/*CLN2*(*TPP1*)/86/M/14 yrs^a^/NA	Positive family history of NCL (<3 yrs)	GDD, seizures (MS, GTCS, drop attacks) blindness	Normal (CB)/cerebral atrophy in MRI (11 yrs)	Compound HTZ known c.509‐1G>C^13^/novel c.1064T>C (p.Leu355Pro) in *CLN2* (*TPP1*)
10/*CLN2*(*TPP1*)/117/M/12yrs^a^/no	Speech delay (3 yrs)	GDD, seizures (AS, MS, TS, AbS), ataxia, behavioral problems	CP & FP (CB)/cerebral atrophy, increased signal in central WM, small thalami in MRI	Compound HTZ known c.509‐1G>C^12^/known c.622C>T (p.Arg208*)^13^ in *CLN2* (*TPP1*)
11φ/*CLN2*(*TPP1*)/170/M/9 yrs^a^/no	Attention deficit (3.5 yrs)	GDD, seizures (MS, GTCS, TS, focal), developmental regression, ASD, behavioral problems, dystonia, ataxia, chorea‐athetosis	CP (SB)/arachnoid cyst in MRI (5 yrs)	Compound HTZ known c.509‐1G>C^12^/known c.229G>C (p.Gly77Arg)^14^ in *CLN2* (*TPP1*)
12φ/*CLN2*(*TPP1*)/171/F/6 yrs^a^ **/**no	Hyperactivity (16 mo)	GDD, seizures (MS, GTCS, TS, focal), dystonia, behavioral problems, ataxia, chorea‐athetosis, dyskinesia, visual impairment	NP/cerebellar atrophy in MRI (3 yrs)	Compound HTZ known c.509‐1G>C^12^/known c.229G>C (p.Gly77Arg)^14^ in *CLN2* (*TPP1*)
13/*CLN3*/42/M/11 yrs/no	Visual problems (4 yrs)	GDD, regression, seizures (GTCS, focal, AbS, TS), behavioral problems	CP & FP (CB)/NP	HMZ known 1.02 kb deletion^15^ in *CLN3*
14$/*CLN3*/2/F/18 yrs/no	Night vision problems (10 yrs)	RP	GROP (CB)/NP	Compound HTZ known c.883G>T (p.Glu295*)^9^/known c.917T>A (p.Leu306His)^9^/in *CLN3*
15$/*CLN3*/44/F/18 yrs/no	Visual problems (10 yrs)	RP, photophobia	NP/NP	Compound HTZ known c.883G>T (p.Glu295*)^9^/known c.917T>A (p.Leu306His)^9^ in *CLN3*
16/*CLN3*/61/M/18 yrs/NA	Visual impairment (<9 yrs)	Cognitive dysfunction, aggressive behavior, blindness, seizures (AbS, AS, GTCS), tremor, ataxia	NP/NP	HMZ known 1.02 kb deletion^15^ in *CLN3*
17/*CLN3*/121/M/18 yrs/yes	Visual impairment (3 yrs)	GDD, regression, cortical blindness, seizures (MS, GTCS, TS, AbS)	NP/cerebral atrophy in MRI (10 yrs)	HMZ novel c.963G>A (p.Trp321*) in *CLN3*
18π/*CLN3*/46/M/18 yrs/yes	Cognitive decline (7 yrs)	GDD, optic atrophy	NP/NP	HMZ known c.1001G>A (p.Arg334His)^15^ in *CLN3*
19π/*CLN3*/144/F/18 yrs/yes	Visual impairment (6 yrs)	Seizures (GTCS), RP, cognitive dysfunction, hypokinesia	CP & FP (CB)/NP	HMZ known c.1001G>A (p.Arg334His)^15^ in *CLN3*
20/*CLN5*/94/F/16 yrs/NA	Balance problems (3.5 yrs)	GDD, spasticity, seizures (MS, AS, AbS, focal), blindness, ataxia	NP/cerebral and cerebellar atrophy, decreased signal in thalami in MRI	HMZ novel c.545T>G (p.Met182Arg) in *CLN5*
21/*CLN6*/15/F/10 yrs/yes	Progressive motor and cognitive dysfunction (4 yrs)	GDD, regression, seizures (GTCS, MS, AbS, focal), optic atrophy	FP (CB)/cerebral, cerebellar atrophy in MRI	HMZ novel c.198+1G>A in *CLN6*
22^b^/*CLN6*/178/M/44 yrs^a^/NA	Lower limb pain (31 yrs)	Depression, dementia, seizures (MS), spasticity, rigidity, ataxia	GROP (SB)/cortical atrophy in CT	HMZ known c.445C>T (p.Arg149Cys)^12^ in *CLN6*
23^b^/*CLN6*/202/F/55 yrs/NA	Lower limb pain (25 yrs)	Dementia, spasticity, rigidity	GROP (SB)/cortical atrophy in CT	HMZ known c.445C>T (p.Arg149Cys)^12^ in *CLN6*
24^b^/*CLN6*/224//F/40 yrs^a^/NA	Epilepsy (20 yrs)	Seizures (MS), dementia, ataxia, spasticity, rigidity	GROP (SB)/cortical atrophy, increased T2 signal in PVWM, decreased signal in thalami in MRI	HMZ known c.445C>T (p.Arg149Cys)^12^ in *CLN6* gene
25/*CLN7*(*MFSD8*)/68/F/14 yrs^a^/no	Balance and speech problems (4 yrs)	GDD, seizures (MS, GTCS, AS, focal), spastic quadriplegia	NP/NP	HMZ known c.754+2T>A^16^ in *CLN7* (*MFSD8*)
26/*CLN7*(*MFSD8*)/78/M/10 yrs/yes	Speech delay (1.25 yrs)	GDD, regression, seizures (MS, GTCS, AS) visual impairment, microcephaly, hypotonia	CP&FP (CB)/cerebral, cerebellar atrophy, small thalami, increased T2 signal PVWM in MRI	HMZ novel c.1241_1242insGAAT (p.Ile414Metfs*109) in *CLN7* (*MFSD8*)
27/*CLN7*(*MFSD8*)/93/F/15 yrs/NA	Cognitive decline regression (5 yrs)	GDD, seizures (GTCS, focal), visual impairment, ataxia	FP (CB)/cerebral, cerebellar atrophy, increased T2 signal PVWM and thalami in MRI	HMZ known c.1394G>A (p.Arg465Gln)^12^ in *CLN7* (*MFSD8*)
28/*CLN7*(*MFSD8*)/100/F/12 yrs/NA	Developmental regression (2.75 yrs)	GDD, regression, seizures (GTCS, MS, AbS, focal), hypertonia, ataxia	NP/cerebellar atrophy, increased T2 signal PVWM in MRI	HMZ known c.881C>A (p.Thr294Lys)^12^ in *CLN7* (*MFSD8*)
29/*CLN7*(*MFSD8*)/116/F/7 yrs 7 mo^a^/NA	Ataxia (3.5 yrs)	GDD, seizures (MS, GTCS, AbS), optic atrophy, ataxia	NP/cerebellar atrophy, increased T2 signal PVWM in MRI	HMZ known c.416G>A (p.Arg139His)^12^ in *CLN7* (*MFSD8*)
30/*CLN7*(*MFSD8*)/129/M/18 yrs/NA	Seizures (7 yrs) Physician diagnosed	GDD, regression, seizures (GTCS, MS, AbS, TS), ataxia, bradykinesia, tremor	Normal (CB)/cerebral atrophy, cerebellar, increased FLAIR signal PVWM in MRI	HMZ novel c.863+4A>G in *CLN7* (*MFSD8*)
31θ/*CLN8*/64/F/18 yrs/yes	Developmental regression (3 yrs 8 mo)	GDD, seizures (MS, GTCS, focal, AS), regression, severe optic atrophy	NP/NP	HMZ known c.473A>G (p.Tyr158Cys)^17^ in *CLN8*
32θ/*CLN8*/85/M/11 yrs^a^/yes	Developmental regression (2.5 yrs)	GDD, spasticity, seizures (GTCS, MS)	CP&FP (CB)/cerebral atrophy, small thalami in MRI	HMZ known c.473A>G (p.Tyr158Cys)^17^ in *CLN8*
33/*CLN8*/73/F/13 yrs/no	Developmental regression (3 yrs)	GDD, seizures (GTCS, MS), visual impairment, dystonia	CP&FP (CB)/cerebellar atrophy, increased T2 signal PVWM in MRI	HMZ known c.473A>G (p.Tyr158Cys)^17^ in *CLN8*

a Deceased age.

b $φπθsiblings.

Abbreviations: AbS, absence seizures; AS, atonic seizures; CB, conjunctival biopsy; CC, corpus callosum; CP, curvilinear profile; CT, computerized tomography; d, day(s); dec, deceased; FP, fingerprint profile; GDD, global developmental delay; GROP, granular osmiophilic profiles; GTCS, generalized tonic‐clonic seizures; HMZ, homozygous; HTZ, heterozygous; mo, months; MRI, magnetic resonance imaging; MS, myoclonic seizures; NCL, neuronal ceroid lipofuscinosis; NP, not performed; PV, periventricular; RP, retinitis pigmentosa; SB, skin biopsy; TS, tonic seizures; WM, white matter; y, year(s).

Conjunctival biopsy or skin biopsy showed lipopigments suggestive of NCL in 15 patients. Granular osmiophilic profile (*CLN1* (*PPT1*) (n = 2), *CLN3* (n = 1) and *CLN6* (n = 3) diseases) and mixed curvilinear and fingerprint profiles (*CLN2* (*TPP1*) (n = 1), *CLN3* (n = 2), *CLN7* (*MFSD8*) (n = 1), and *CLN8* (n = 2) diseases) were the most common lipopigments. Three patients had normal histopathology.

**Figure 1:**
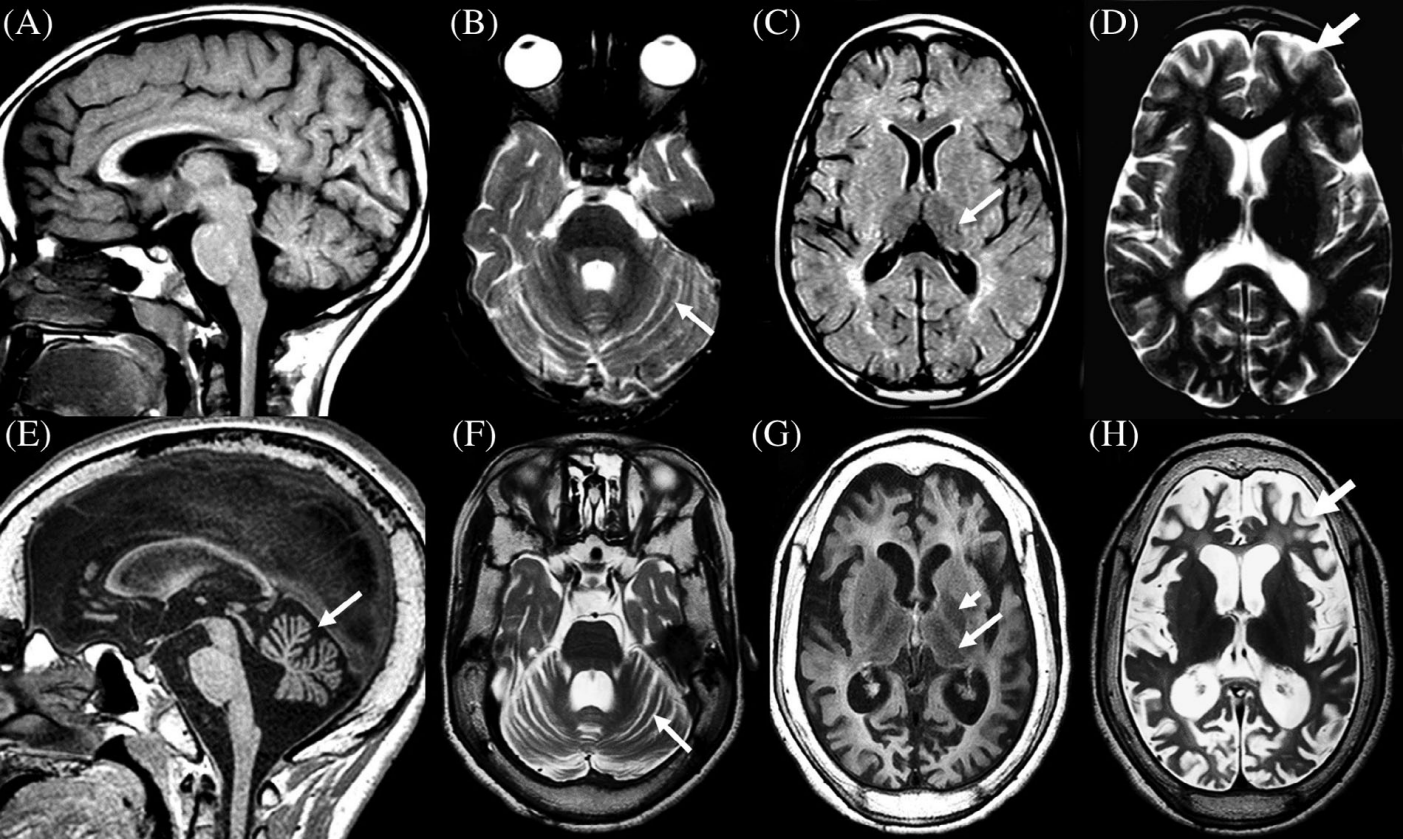
Two brain MRIs and disease progression in a patient with *CLN1 (PPT1*) associated disease (patient 5, ID#76 in Table 1). Top row 4y2m (A‐D): Sagittal T1 weighted image (A) demonstrates a normal corpus callosum, brainstem and vermis. Axial T2 weighted image (B) reveals mild widening of the interfoliate sulci (arrow) of the cerebellar hemispheres. Axial FLAIR image (C) shows dark signal intensity of the thalami (arrow) and increased signal of the periatrial white matter. Mild widening of the supratentorial sulci is noted (arrow) on T2 weighted axial image (D). Bottom row 7y8m (E‐H): Marked thickening of the calvarium is shown on all images. There is volume loss of the corpus callosum and moderate atrophy of the vermis (arrow) on T1 weighted sagittal image (E). Progressive widening of the interfoliate sulci (arrow) of the cerebellar hemispheres is shown on axial T2 weighted image (F). There is persistent loss of thalamic signal and volume (arrow) on axial FLAIR image (G). On the same image, loss of globus pallidus signal is present (short arrow) and the white matter is diffusely abnormally increased in signal. Marked widening of the supratentorial sulci (arrow) and moderate ventricular enlargement is noted on axial T2 weighted image (H), reflecting both central and peripheral volume loss

Twenty‐two patients had brain MRI and two patients had brain CT. The most common brain MRI feature was diffuse cerebral and/or cerebellar atrophy in 21 patients. Brain MRI of a patient with *CLN1* (*PPT1*) disease and progression of MRI features are depicted in Figure 1.

In silico analysis and ACMG variant classification of all variants are listed in Table S1. There were 52 different variants in seven genes in 91 patients including 11 novel and 41 known variants: *CLN1* (*PPT1*) variants n = 8^10^, ^11^, ^12^, ^18^, ^19^; *CLN2* (*TPP1*) variants n = 11^12^, ^13^, ^14^, ^20^, ^21^, ^22^, ^23^; *CLN3* variants n = 11^9^, ^12^, ^15^, ^24^, ^25^; *CLN5* variants n = 5^12^, ^21^, ^26^; *CLN6* variants n = 7^12^, ^21^, ^27^, ^28^; *CLN7* (*MFSD8*) variants n = 8^12^, ^16^, ^29^; and *CLN8* variants n = 2.^17^, ^30^ The number of missense and truncating variants for each gene is depicted in Figure S3. The most common variant type was truncating in *CLN3*, *CLN5*, and *CLN6*. According to ACMG variant classification, 25 variants were classified as pathogenic, and 24 variants were classified as likely pathogenic. Three variants were classified as variant of unknown significance including two novel (c.545T>G; p.Met182Arg in *CLN5* and c.863+4A>G in *CLN7)* and one known (c.445C>T; p.Arg149Cys in *CLN6* [12]). All of those patients were listed in Table 1 for their clinical information with a molecular genetic confirmation of NCL. We did not have any clinical information for three patients with five variants (homozygous or compound heterozygous, one known pathogenic and four novel variants of unknown significance) in *CLN2*, *CLN6*, and *CLN8* and did not include them into our list of patients with molecular genetic confirmation of NCL (Table S2). Four missense variants were located in the last two nucleotides of an exon and known to affect splicing: c.229G>C (p.Gly77Arg) and c.1266G>C (p.Gln422His) in *CLN2* (*TPP1*), and c.553G>A (Val185Ile) and c.697A>G (p.Arg233Gly) in *CLN7* (*MFSD8*). The intronic variants were analyzed and have been predicted to affect splicing including c.906+6T>G variant in *CLN3* (MaxENT score of −36.7%, NNSplice prediction of −81.6%, Genesplicer prediction of −62.5%); c.320+5G>C variant in *CLN5* (MaxENT prediction of −82.2%, NNSplice prediction of −90.7%, Genesplicer prediction of −58.0%); and c.863+4A>G variant in *CLN7* (*MFSD8*) (MaxENT prediction of −32.0%, NNSplice prediction of −40.2%, SSF prediction of −11.7%). None of the novel variants were found in dbSNP database as polymorphisms. The novel missense variants were highly conserved across species, except one variant which was moderately conserved (c.223A>C; p.Thr75Pro),^11^ and reported to be disease causing in in silico analysis.

We had clinical features and histopathology and neuroimaging results of 76 out of 343 symptomatic patients with no molecular genetic diagnosis of NCL at our institution. Distribution of clinical features and histopathology and neuroimaging results of 33 patients with molecular genetic diagnosis of NCL and 76 patients with no molecular genetic diagnosis of NCL are summarized in Table 2. The history of regression, cognitive decline and visual impairment and presence of cerebral and cerebellar atrophy in brain MRI and presence of lipopigments in biopsy histopathology were significantly different in patients with molecular genetic diagnosis of NCL (Fisher's exact test *P* < .05). Hypotonia, infantile spasms, normal biopsy histopathology and white matter abnormalities in brain MRI were significantly different in patients with no molecular genetic diagnosis of NCL (Fisher's exact test *P* < .05).

**Table 2:** Distribution of clinical features, neuroimaging features, and histopathology results of patients with and without molecular genetic diagnosis of NCL are listed. Fisher's exact test was applied for the comparison of proportions for each finding between two groups and are listed

Clinical features and investigations	Number of patients with molecular genetic diagnosis of NCL (percentage) (n = 33)	Number of patients with no molecular genetic diagnosis of NCL (percentage) (n = 76)	Fisher's exact test *P* value
*Neurodevelopment and neurological features*			
GDD	23 (69.6%)	56 (73.7%)	.8158
Regression or cognitive decline	19 (57.5)	15 (19.7%)	.0017
Hypotonia	1 (3%)	22 (28.9%)	.0017
Hypertonia	2 (6%)	13 (17.1%)	.224
*Seizures*	27 (81.8%)	59 (77.6%)	.7993
GTCS	21 (63.6%)	23 (30.2%)	.0015
Myoclonic	18 (54.5%)	20 (26.3%)	.008
Absence	11 (33.3%)	17 (22.3%)	.2417
Infantile spasms	0 (0%)	12 (15.7%)	.0165
Partial Seizures	11 (33.3%)	10 (13.1%)	.0188
*Visual impairment*	22 (66.6%)	13 (17.1%)	<.0001
*Movement disorder*	19 (57.5%)	33 (43.4%)	.2125
Ataxia	13 (39.3%)	15 (19.7%)	.0545
Dystonia	3 (9.1%)	0 (0%)	.026
Tremor	3 (9.1%)	7 (9.2%)	1
*Behavioral problems*	7 (21.2%)	13 (17.1%)	.6007
*Failure to thrive*	1 (3%)	8 (10.5%)	.2722
*Histopathology*	18	19	
Normal	3 (16.6%)	19 (100%)	<.0001
Granular osmiophilic inclusions	6	0	
Finger print	2	0	
Curvilinear and fingerprint profiles	6	0	
*Brain MRI features*	22	67	
Cerebral and/or cerebellar atrophy	21 (95.4%)	37 (55.2%)	.0005
Abnormal T2/FLAIR signal intensities	15 (68.2%)	18 (26.8%)	.0008
Corpus callosum abnormalities	1 (4.5%)	17 (25.3%)	.0362
Delayed myelination	0 (0%)	12 (17.9%)	.0077
Hypomyelination	0 (0%)	4 (5.9%)	.2991

Abbreviations: GDD, global developmental delay; GTCS, generalized tonic‐clonic seizures; MRI, magnetic resonance imaging.

Twenty‐four out of 76 symptomatic patients were heterozygotes for 22 variants including five known pathogenic and three likely pathogenic (two known and one novel) variants. All those variants and their ACMG variant classification are listed in Table S3.

## 4 DISCUSSION

We report 27% diagnostic yield of direct Sanger sequencing of the *CLN* genes (22% in families with more than one symptomatic child). *CLN2* (*TPP1*) disease was the most common subtype (36%) and *CLN3* disease was the second most common subtype (19%) of NCL in our study. Distribution of NCL in the UCL NCL Resource Patient Database (http://www.ucl.ac.uk/ncl/) is depicted in Figure S4. We report detailed clinical features of 33 NCL patients and 11 novel variants in the *CLN* genes. Presence of developmental regression, cognitive decline and progressive visual impairment in the history, and cerebral and cerebellar atrophy in brain MRI are significant clinical and neuroimaging denominators and should warrant physicians to investigate NCL using direct Sanger sequencing of the *CLN* genes.

In patients with neurodegenerative disorders, skin biopsy was applied to identify underlying causes in a few studies. The diagnostic yield of NCL based on the lipopigments was <10% in all of those studies (Williams et al., 2006).^31^, ^32^, ^33^, ^34^, ^35^, ^36^ In one of those studies, 143 patients had axillary skin biopsy for the evaluation of metabolic disease. Only one patient had curvilinear profile and was morphologically diagnosed with NCL. Morphological diagnostic yield of skin biopsy was 0.7% in that study.^31^ In another study, 184 pediatric patients underwent skin biopsy including 139 patients with progressive encephalopathies and 45 patients with static encephalopathies. Morphological diagnosis of NCL was confirmed in 6.5% (12 out of 184) patients.^32^ In 2013, the experts recommended an algorithm for the diagnostic investigations of NCL in patients with suggestive symptoms including enzyme tests and biopsies to investigate for lipopigments to perform economical and adequate diagnostic investigations for the molecular confirmation of NCL.^3^ The molecular genetic tests identify variants in NCL genes in more than 90% of the patients with phenotypic features suggestive of NCL.^36^ To the best of our knowledge, there are no studies that reported the diagnostic yield of next generation sequencing or whole exome sequencing for NCL genetic diagnosis.

Morphological diagnosis of *CLN1* (*PPT1*) (granular osmiophilic profile), and *CLN2* (*TPP1*) (curvilinear profile) diseases have been reported in the majority of patients with the genotypic confirmation.^37^ Mixed lipopigment profiles were also reported in *CLN1* (*PPT1*) (15%) and *CLN2* (*TPP1*) (20%) diseases in the UCL NCL Resource Patient Database. In our study, we found mixed profiles in 33% (6 out of 18 patients with biopsy) and normal histopathology in 17% of the patients. Genotype and morphotype correlation was present in 17% of the patients (*CLN1* n = 2 and *CLN2* n = 1) with a confirmed molecular genetic diagnosis of NCL in our study. In centers, where molecular genetic tests are not readily available or funded through health care system, in the presence of suggestive history and brain MRI features, conjunctival or skin biopsy histopathology will likely help clinicians to guide them for direct Sanger sequencing of the *CLN* genes. Normal histopathology and normal NCL genetic test results are sufficient to exclude NCL.

In the UCL NCL Resource Patient Database, *CLN1* (*PPT1*) disease was juvenile onset (≥2 years of age) in 27% of the patients, whereas in our study, 71% of the patients had juvenile disease onset (≥4 years). Interestingly, in three out of five patients with juvenile onset *CLN1 (PPT1)* disease, vison problems were the presenting symptom in our study. In the UCL NCL Resource Patient Database, *CLN6* disease was adult onset in 12% of the patients, whereas in our study three out of four (75%) patients with *CLN6* disease had adult onset. Interestingly, in two of those patients, lower limb spasticity was the presenting symptom. The phenotypic spectrum is highly variable in *CLN1* (*PPT1*) and *CLN6* diseases and application of targeted next generation sequencing panels for seizures, intellectual disability or retinopathy, or whole exome sequencing will likely identify more patients with nonclassical NCL phenotypes. In patients with retinopathy and spastic paraplegia, targeted next generation sequencing panels should also include all *CLN* genes due to variable phenotype within the same genotype.

Our Research Ethics Board approved the study with the condition of two consents signed by parents or institutional Research Ethics Board approvals at each institution. The majority of clinicians did not apply to their Research Ethics Board due to single patient or small number of patients at their institution. The majority of the clinicians did not want to contact parents to provide study information, as their children had passed away due to NCL or clinical contact was lost. Due to these, we received phenotypic information in 9.4% of NCL patients (6 out of 64 patients) outside of our Institution. Additionally, we were not allowed to collect any other genetic diagnoses other than NCL in our database as per request of our Research Ethics Board. We were also requested not to report any other genetic diagnoses in patients with no molecular genetic diagnosis of NCL in the manuscript. For these reasons, we do not have any data for other genetic diagnoses in the group of 76 symptomatic patients with no molecular genetic diagnosis of NCL at our Institution. The remaining 176 symptomatic patients were referred from outside of our institution that we did not have any clinical information for those. A follow‐up multicenter study would be interesting to identify the diagnostic yield of targeted next generation sequencing panels or whole exome sequencing for NCL. It is not certain if the diagnostic yield of next generation sequencing would be higher than our study results.

The most commonly reported variant in *CLN1* (*PPT1*) was c.451C>T (p.Arg151*) in the UCL NCL Resource Mutation Database, which is a panethnic variant. This variant resulted in phenotypes ranging from infantile onset to adult onset either compound heterozygous or homozygous. We also had similar results in our small cohort of patients with *CLN1* (*PPT1*) disease for this common variant.

In conclusion, we report 27% diagnostic yield of direct Sanger sequencing of the *CLN* genes in symptomatic patients (22% in families with more than one affected child). Developmental regression, cognitive decline, visual impairment and cerebral and/or cerebellar atrophy in brain MRI are significant clinical and neuroimaging denominators to include NCL in the differential diagnosis. Juvenile onset *CLN1* (*PPT1*) and adult onset *CLN6* disease phenotypes were the most common nonclassical phenotypes in our study. Whole exome sequencing or targeted next generation sequencing panels for epilepsy, intellectual disability and retinopathy will likely identify more patients with nonclassical phenotypes of NCL and should be the first line investigations in patients with a history of progressive neurodegenerative encephalopathy.

## AUTHOR CONTRIBUTIONS

A.J. reviewed charts, generated database, drafted the manuscript, conducted the work, and involved in the approval of the final version. D.M. generated database from the molecular genetics laboratory, reviewed all variants for in silico analysis and classified all variants based on the ACMG variant classification guidelines, and involved in the approval of the final version. S.B. reviewed patient MRIs and provided figure and information, and involved in the approval of the final version. S.D., J.M., A.N., C.P. provided cases from their centers and involved in the approval of the final version. L.K. generated database from the molecular genetics laboratory and involved in the approval of the final version. S.M‐A involved in planning, applying, and receiving funding; conduct, drafting, and revising the manuscript; and approval of the final version.

## CONFLICT OF INTEREST

The authors declare that they have no conflict of interest.

## ETHICAL APPROVAL AND PATIENT CONSENT

Institutional Research Ethics Board (Approval#1000059020) approved this study.

Informed consent was obtained for patients outside of our institution.

All procedures followed were in accordance with the ethical standards of the responsible committee on human experimentation (institutional and national) and with the Helsinki Declaration of 1975, as revised in 2000 (5).

Proof that informed consent was obtained is available upon request.

There is no identifying information about patients included in the article.

This article does not contain any studies with animal subjects performed by the any of the authors.

## Supporting information


**Table S1** In silico analysis of variants in NCL genes identified in patients is listed.
**Table S2:** In silico analysis of compound heterozygous or homozygous variants of unknown significance in NCL genes, identified in patients with no clinical information are listed.
**Table S3:** In symptomatic patients, number of heterozygotes for each NCL gene and variants in each NCL gene are listed.

**Figure S1** The distribution of number of patients in each NCL gene, identified in this study.

**Figure S2** Survival of 11 patients.

**Figure S3** Distribution of missense and truncating variants for each NCL gene in our study.

**Figure S4** The distribution of number of patients in each NCL gene in the UCL NCL Resource Patient Database.

## Data Availability

The authors confirm that the part of the data supporting the findings of this study is available within the article (and/or) its supplementary material based on the Research Ethics Board approvals. The data that support the findings of this study analysis are available on request from the corresponding author. The data are not publicly available due to data containing research participants' information could compromise the privacy of research participants.
