# Supplementary material for: High diagnostic yield of direct Sanger sequencing in the diagnosis of neuronal ceroid lipofuscinoses
Source: JIMD Rep. 2019 Sep 3;50(1):20–30. doi: 10.1002/jmd2.12057 (PMC6850977; doi:10.1002/jmd2.12057)
Supplement: Supplementary file 1 — Table S1 In silico analysis of variants in NCL genes identified in patients is listed. Table S2: In silico analysis of compound heterozygous or homozygous variants of unknown significance in NCL genes, identified in patients with no clinical information are listed. Table S3: In symptomatic patients, number of heterozygotes for each NCL gene and variants in each NCL gene are listed. [file JMD2-50-20-s001.docx]

**Supplemental Tables**

**Supplementary Table 1:** *In silico* analysis of variants in NCL genes identified in patients are listed in Supplementary Table 1.

| **Gene** | **Variant** | **SIFT** | **MutTaster** | **PolyPhen-2** | **Conservation in species** | **gnomAD allele count in allele number** | **Variant Classification** |
| --- | --- | --- | --- | --- | --- | --- | --- |
| *CLN1* (*PPT1*)  NM_000310.3 | c.29T>A (p.Leu10*) (Das et al., 1998) | NA | NA | NA | NA | 20 in 276316 | Pathogenic  (PVS1, PS3, PS4, PM2, PP5) |
|  | c.223A>C (p.Thr75Pro) (Waliany et al., 2000) | Tolerated | Disease causing | Benign | 6 out of 13 | 4 in 246234 | Pathogenic  (PS3, PS4, PM2, PP5) |
|  | c.289_290del (p.Gln97Glyfs*4) | NA | NA | NA | NA | NA | Pathogenic  (PVS1, PM2, PM3) |
|  | c.451C>T (p.Arg151*) (Das et al., 1998) | NA | NA | NA | NA | 69 in 277130 | Pathogenic  (PVS1, PS3, PS4) |
|  | c.490C>T (p.Arg164*) (Kousi et al., 2012) | NA | NA | NA | NA | 2 in 246202 | Pathogenic (PVS1, PM2, PP5) |
|  | c.541G>A (Val181Met) (Kousi et al., 2012) | Deleterious | Disease causing | Probably Damaging | 12 out of 12 | 24 in 277014 | Likely pathogenic  (PS4, PM1, PM2, PP3) |
|  | c.541G>T (p.Val181Leu) (Kousi et al., 2012) | Deleterious | Disease Causing | Benign | 12 out of 12 | 2 in 246126 | Likely pathogenic  (PM1, PM2, PM3, PP5) |
|  | c.674T>C (p.Phe225Ser) (Mole et al., 2001) | Deleterious | Disease Causing | Probably Damaging | 12 out of 13 | 1 in 246180 | Likely pathogenic  (PM1, PM2, PP3, PP5) |
| *CLN2* (*TPP1*)  NM_000391.3 | c.229G>C (p.Gly77Arg) (Walus et al., 2010) | Deleterious | Disease Causing | Probably Damaging | 11 out of 11 | NA | Likely pathogenic  (PM2, PM5, PP3, PP5) |
|  | c.509-1G>C  (r.spl?) (Kousi et al., 2012) | NA | NA | NA | NA | 113 in 277058 | Pathogenic  (PVS1, PS4, PM3, PP5) |
|  | c.622C>T (p.Arg208*) (Elleder et al., 2008) | NA | NA | NA | NA | 73 in 276920 | Pathogenic  (PVS1, PS3, PS4, PP5) |
|  | c.756_761delinsCCGTGT (p.His253_Gln254delinsArgVal) | NA | NA | NA | NA | NA | Likely pathogenic  (PM1, PM2, PM4) |
|  | c.775delC  (p.Arg259Valfs*17) (Goldberg-Stern et al., 2009) | NA | NA | NA | NA | NA | Likely pathogenic  (PVS1, PM2) |
|  | c.851G>T (p.Gly284Val) (Zhong et al., 2000) | Deleterious | Disease Causing | Probably Damaging | 10 out of 10 | 2 in 246248 | Pathogenic  (PS3, PS4, PM2, PP3, PP5) |
|  | c.1064T>C (p.Leu355Pro) (Kousi et al., 2012) | Deleterious | Disease Causing | Probably Damaging | 10 out of 11 | NA | Likely pathogenic  (PS3, PM1, PM2, PP3) |
|  | c.1093T>C (p.Cys365Arg) (Sleat et al., 1999) | Deleterious | Disease Causing | Probably Damaging | 11 out of 11 | 1 in 30952 | Pathogenic  (PS3, PM1, PM2, PP3, PP5) |
|  | c.1266G>C (p.Gln422His) (Sleat et al., 1999) | Deleterious | Disease Causing | Probably Damaging | 11 out of 11 | 5 in 246258 | Pathogenic  (PS4, PM1, PM2, PM3, PP3) |
|  | c.1424delC  (p.Ser475Trpfs*13) (Moore et al., 2008) | NA | NA | NA | NA | NA | Pathogenic  (PVS1, PS4, PM1, PM2) |
|  | c.1444G>A (p.Gly482Arg) (Kousi et al., 2009) | Deleterious | Disease causing | Probably Damaging | 8 out of 11 | NA | Likely pathogenic  (PM1, PM2, PM5, PP3) |
| *CLN3*  NM_001042432.1 | 1.02Kb del (Munroe et al., 1997) | NA | NA | NA | NA | NA | Pathogenic (PS2, PS4, PM3) |
|  | c.461-1G>C  (r.spl?) (Kousi et al., 2012) | NA | NA | NA | NA | NA | Likely pathogenic  (PVS1, PM2) |
|  | c.622dupT (p.Ser208Phefs*28) (Pérez-Poyato et al., 2011) | NA | NA | NA | NA | 3 in 243678 | Pathogenic (PVS1, PS4, PM2, PP5) |
|  | c.883G>T (p.Glu295*) (Ku et al., 2017) | NA | NA | NA | NA | NA | Pathogenic  (PVS1, PS4, PM2, PP5) |
|  | c.906+6T>G  (r.spl?) (Lau et al., 2018) | NA | NA | NA | NA | 1 in 246242 | Likely pathogenic  (PS4, PM2, PP3) |
|  | c.917T>A (p.Leu306His) (Ku et al., 2017) | Deleterious | Disease Causing | Probably Damaging | 9 out of 12 | NA | Likely pathogenic  (PM1, PM2, PM3, PP3) |
|  | c.944dupA (p.His315Glnfs*67) (Munroe et al., 1997) | NA | NA | NA | NA | 1 in 245564 | Pathogenic  (PVS1, PS4, PM2) |
|  | c.963G>A (p.Trp321*) | NA | NA | NA | NA | NA | Likely pathogenic  (PVS1, PM2) |
|  | c.1000C>T (p.Arg334Cys) (Munroe et al., 1997) | Deleterious | Disease Causing | Probably Damaging | 12 out of 12 | 1 in 244340 | Likely pathogenic  (PS4, PM1, PM2, PP3) |
|  | c.1001G>A (p.Arg334His) (Munroe et al., 1997) | Deleterious | Disease Causing | Probably Damaging | 12 out of 12 | 7 in 275064 | Likely pathogenic  (PS4, PM1, PM2, PP3) |
|  | c.1004C>A (p.Ser335Tyr) | Deleterious | Disease Causing | Probably damaging | 11 out of 12 | NA | Pathogenic  (PS3, PS4, PM1, PM2, PP3) |
| *CLN5*  NM_006493.2 | c.225G>A (p.Trp75*) (Kousi et al., 2012) | NA | NA | NA | NA | 6 in 221684 | Pathogenic  (PVS1, PM2, PM3, PP5) |
|  | c.545T>G (p.Met182Arg) | Deleterious | Disease Causing | Benign | 9 out of 12 | 1 in 246264 | VUS  (PM2) |
|  | c.669dupC (p.Trp224Leufs*30) (Kousi et al., 2012) | NA | NA | NA | NA | 8 in 246246 | Pathogenic  (PVS1, PS3, PS4, PM2, PM3) |
|  | c.1054G>T (p.Glu352*) (Moore et al., 2008) | NA | NA | NA | NA | NA | Pathogenic  (PVS1, PM2, PP5) |
|  | c.613C>T (p.Pro205Ser) (Al-Kowari et al., 2011) | Deleterious | Disease causing | Probably Damaging | 12 out of 12 | NA | Likely pathogenic  (PS4, PM2, PP3, PP5) |
| *CLN6*  NM_017882.2 | c.198+1G>A  (r.spl?) | NA | NA | NA | NA | NA | Pathogenic  (PVS1, PM2, PP5) |
|  | c.218G>A (p.Trp73*) | NA | NA | NA | NA | NA | Likely pathogenic  (PVS1, PM2) |
|  | c.268_271dupAACG (p.Val91Glufs*42) (Munroe et al., 1997) | NA | NA | NA | NA | NA | Pathogenic  (PVS1, PM2, PP5) |
|  | c.316dupC (p.Arg106Profs*26) (Guerreiro et al., 2013) | NA | NA | NA | NA | 6 in 245396 | Pathogenic  (PVS1, PS4, PM2, PP5) |
|  | c.395_396delCT (p.Ser132Cysfs*18) (Guerreiro et al., 2013) | NA | NA | NA | NA | 2 in 246118 | Pathogenic  (PVS1, PM2, PM3) |
|  | c.445C>T (p.Arg149Cys) (Kousi et al., 2012) | Deleterious | Disease Causing | Probably Damaging | 11 out of 11 | 16 in 276954 | VUS  (PM2, PP3) |
|  | c.461_463delTCA (p.Ile154del) | NA | NA | NA | NA | 1 in 245968 | Likely pathogenic  (PS4, PM2, PM4, PP5) |
| *CLN7* (*MFSD8*)  NM_152778.2 | c.416G>A (p.Arg139His) (Kousi et al., 2012) | Deleterious | Disease Causing | Probably Damaging | 12 out of 12 | 1 in 246012 | Pathogenic  (PS4, PM1, PM2, PP3, PP5) |
|  | c.697A>G (p.Arg233Gly) (Siintola et al., 2007) | Tolerated | Disease causing | Probably Damaging | 11 out of 13 | NA | Likely pathogenic  (PS4, PM1, PM2) |
|  | c.754+2T>A  (r.spl?) (Siintola et al., 2007) | NA | NA | NA | NA | 3 in 276446 | Pathogenic  (PVS1, PS4, PM2) |
|  | c.863+4A>G  (r.spl?) | NA | NA | NA | NA | 5 in 276802 | VUS  (PM2, PP3) |
|  | c.881C>A (p.Thr294Lys) (Kousi et al., 2012) | Deleterious | Disease Causing | Possibly Damaging | 12 out of 13 | 1 in 244390 | Likely pathogenic  (PS4, PM2, PP3, PM5) |
|  | c.1241_1242insGAAT  (p.Ile414Metfs*109) | NA | NA | NA | NA | NA | Likely pathogenic  (PVS1, PM2) |
|  | c.1394G>A (p.Arg465Gln) (Kousi et al., 2012) | Deleterious | Disease Causing | Probably Damaging | 13 out of 13 | NA | Likely pathogenic  (PS4, PM1, PM2, PP3) |
|  | Exon 4 deletion identified by QPCR | NA | NA | NA | NA | NA | Likely pathogenic (PVS1, PM2) |
| *CLN8*  NM_018941.3 | c.473A>G (p.Tyr158Cys) (Gao et al., 2018) | Deleterious | Disease Causing | Probably Damaging | 10 out of 11 | 3 in 246256 | Likely pathogenic  (PS4, PM2, PP3, PP5) |
|  | c.792C>G (p.Asn264Lys) (Kohan et al., 2015) | Deleterious | Disease Causing | Possibly Damaging | 10 out of 11 | 1 in 31406 | Likely pathogenic  (PS4, PM2, PP3, PP5) |

**Abbreviations (listed alphabetically):** gnomAD= Genome Aggregation Database (gnomAD); MutTaster= Mutation Taster; NA= not available; PolyPhen-2=Polymorphism Phenotyping V2; SIFT= Sorting Intolerant From Tolerant; VUS=variant of known significance.

**Supplementary Table 2:** *In silico* analysis of compound heterozygous or homozygous variants of unknown significance in NCL genes, identified in patients with no clinical information are listed in Supplementary Table 2.

| **Patients** | **Gene** | **Variant** | **SIFT** | **MutTaster** | **PolyPhen-2** | **Conservation in Species** | **gnomAD allele count in allele number** | **Variant Classification** |
| --- | --- | --- | --- | --- | --- | --- | --- | --- |
| 1 | *CLN2* (*TPP1*)  NM_000391.3 | c.509-1G>C (r.spl?) (Kousi et al., 2012) | NA | NA | NA | NA | 113 in 277058 | Pathogenic (PVS1, PS4, PM3, PP5) |
|  |  | c.1603G>A (p.Gly535Ser) | Deleterious | Disease causing | Probably damaging | 10 out of 11 | NA | VUS (PM2, PP3) |
| 2 | *CLN6*  NM_017882.2 | c.307C>T (p.Arg103Trp) | Deleterious | Disease causing | Probably damaging | 11 out of 11 | 12 in 281500 | VUS (PM2, PP3) |
|  |  | c.209C>T (p.Pro70Leu) | Deleterious | Disease causing | Probably damaging | 10 out of 11 | NA | VUS (PM2, PP3) |
| 3 | *CLN8*  NM_018941.3 | HMZ c.601T>A (p.Phe201Ile) | Deleterious | Disease causing | Probably damaging | 10 out of 11 | NA | VUS  (PM2, PP3) |

**Abbreviations (listed alphabetically):** gnomAD= Genome Aggregation Database (gnomAD); MutTaster= Mutation Taster; NA= not available; PolyPhen-2=Polymorphism Phenotyping V2; SIFT= Sorting Intolerant From Tolerant; VUS=variant of known significance.

**Supplementary Table 3** In symptomatic patients, number of heterozygotes for each NCL gene and variants in each NCL gene are listed in Supplementary Table 3.

| **Gene**  **(number of heterozygotes in symptomatic patients)** | **Variants** | **Variant Classification** |
| --- | --- | --- |
| *CLN1* (*PPT1*) NM_000310.3  (2) | c.29T>A (p.Leu10*) (Das et al., 1998) | Pathogenic (See Table S1) |
|  | c.451C>T (p.Arg151*) (Das et al., 1998) | Pathogenic (See Table S1) |
| *CLN2* (*TPP1*) NM_000391.3  (5) | c.509-1G>C (r.spl?) (Kousi et al., 2012) | Pathogenic (See Table S1) |
|  | c.622C>T (p.Arg208*) (Elleder et al., 2008) | Pathogenic (See Table S1) |
|  | c.1444G>A (p.Gly482Arg) (Kousi et al., 2009) | Likely pathogenic (See Table S1) |
|  | c.1603G>A (p.Gly535Ser) | VUS (See Table S2) |
|  | c.665A>G (p.Asn222Ser) | VUS (PM2) |
| *CLN3* NM_001042432.1  (2) | c.242C>T(p.Pro81Leu) | VUS |
|  | c.883G>T(p.Glu295*) (Ku et al., 2017) | Pathogenic (See Table S1) |
| *CLN5* NM_006493.2  (3) | c.174G>A (p.Gln58Gln) | VUS (PM2, BP7) |
|  | c.253G>C (p.Ala85Pro) | VUS (BS2) |
|  | c.1166A>C (p.Lys389Thr) | VUS (PM2) |
| *CLN6* NM_017882.2  (4) | c.307C>T (p.Arg103Trp) | VUS (PM2, PP3) |
|  | c.209C>T (p.Pro70Leu) | VUS (PM2, PP3) |
|  | c.446G>A (p.Arg149His) | VUS (PM2, PP3) |
| *CLN7* (*MFSD8*) NM_152778.2  (5) | c.16A>C (p.Asn6His) | VUS (PM2, BP4) |
|  | c.593_594delTG (p.Val198Aspfs*6) | Likely pathogenic  (PVS1, PM2) |
|  | c.1171A>G (p.Met391Val) | VUS (PM2, BP4) |
|  | c.1273C>G (p.Leu425Val) | VUS (PM2) |
| *CLN8* NM_018941.3  (2) | c.374A>G (p.Asn125Ser) | VUS |
|  | c.792C>G (p.Asn264Lys) (Kohan et al., 2015) | Likely pathogenic (See Table S1) |
| *CLN10 (CTSD)* NM_001909.4  (1) | c.970G>A (p.Glu324Lys) | VUS (PM2, PP3) |

**Abbreviations:** VUS=variant of known significance.
