# Supplementary figures and images for: High diagnostic yield of direct Sanger sequencing in the diagnosis of neuronal ceroid lipofuscinoses
Source: JIMD Rep. 2019 Sep 3;50(1):20–30. doi: 10.1002/jmd2.12057 (PMC6850977; doi:10.1002/jmd2.12057)

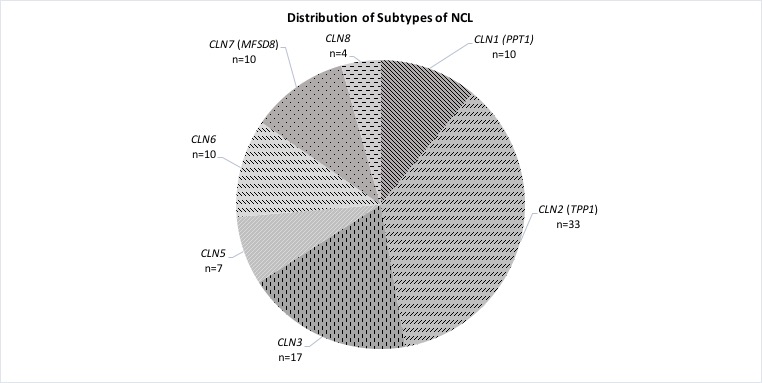

Supplement: Supplementary file 2 — Figure S1 The distribution of number of patients in each NCL gene, identified in this study. [file JMD2-50-20-s002.tiff]

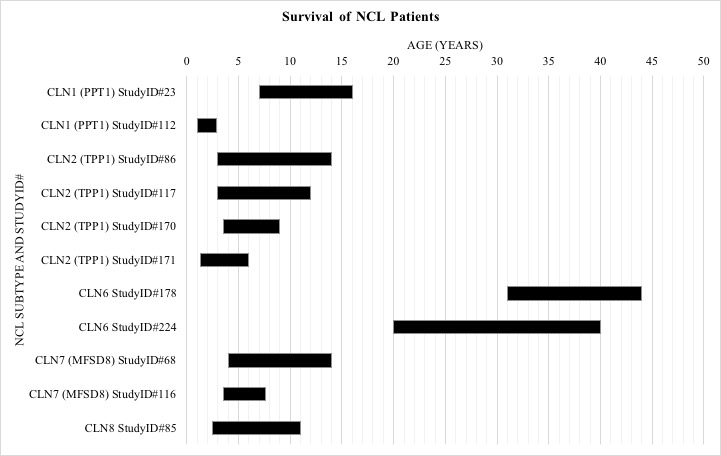

Supplement: Supplementary file 3 — Figure S2 Survival of 11 patients. [file JMD2-50-20-s003.tiff]

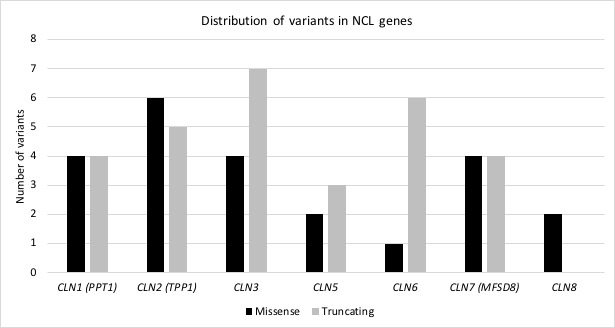

Supplement: Supplementary file 4 — Figure S3 Distribution of missense and truncating variants for each NCL gene in our study. [file JMD2-50-20-s004.tiff]

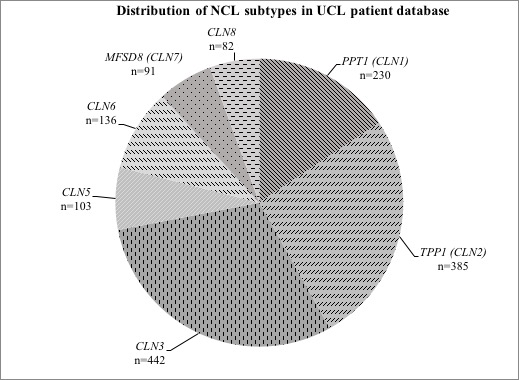

Supplement: Supplementary file 5 — Figure S4 The distribution of number of patients in each NCL gene in the UCL NCL Resource Patient Database. [file JMD2-50-20-s005.tiff]
